# Feasibility of establishing an HIV vaccine preparedness cohort in a population of the Uganda Police Force: Lessons learnt from a prospective study

**DOI:** 10.1371/journal.pone.0231640

**Published:** 2020-04-17

**Authors:** Ubaldo Mushabe Bahemuka, Andrew Abaasa, Janet Seeley, Moses Byaruhanga, Anatoli Kamali, Philippe Mayaud, Monica Kuteesa

**Affiliations:** 1 Medical Research Council/Uganda Virus Research Institute Uganda Research Unit & London School of Hygiene and Tropical Medicine (MRC/UVRI & LSHTM) Uganda Research Unit, Entebbe, Uganda; 2 London School of Hygiene and Tropical Medicine, London, United Kingdom; 3 Uganda Police Force, Directorate of Health Services, Kampala, Uganda; 4 International AIDS Vaccine Initiative, New York, New York, United States of America; The Chinese University of Hong Kong, HONG KONG

## Abstract

**Background:**

Members of uniformed armed forces are considered to be at high risk for HIV infection and have been proposed as suitable candidates for participation in HIV intervention studies. We report on the feasibility of recruitment and follow up of individuals from the community of the Uganda Police Force (UPF) for an HIV vaccine preparedness study.

**Methods:**

HIV-negative volunteers aged 18–49 years, were identified from UPF facilities situated in Kampala and Wakiso districts through community HIV counselling and testing. Potential volunteers were referred to the study clinic for screening, enrolment and quarterly visits for one year. HIV incidence, retention rates were estimated and expressed as cases per 100 person years of observation (PYO). Rate ratios were used to determine factors associated with retention using Poisson regression models.

**Results:**

We screened 560 to enroll 500 volunteers between November 2015 and May 2016. One HIV seroconversion occurred among 431 PYO, for an incidence rate of 0.23/100 PYO (95% confidence interval [CI]: 0.03–1.64). Overall, retention rate was 87% at one year, and this was independently associated with residence duration (compared to <1 year, 1 to 5 years adjusted rate ratio (aRR) = 1.19, 95%CI: 1.00–1.44); and >5 years aRR = 1.34, 95%CI: 0.95–1.37); absence of genital discharge in the last 3 months (aRR = 1.97, 95% CI: 1.38–2.83, absence of genital ulcers (aRR = 1.90, 95%CI: 1.26–2.87, reporting of new sexual partner in the last month (aRR = 0.57, 95%CI: 0.45–0.71, being away from home for more than two nights (aRR = 1.27, 95%CI: 1.04–1.56, compared to those who had not travelled) and absence of knowledge on HIV prevention (aRR = 2.67, 95%CI: 1.62–4.39).

**Conclusions:**

While our study demonstrates the feasibility of recruiting and retaining individuals from the UPF for HIV research, we did observe lower than anticipated HIV incidence, perhaps because individuals at lower risk of HIV infection may have been the first to come forward to participate or participants followed HIV risk reduction measures. Our findings suggest lessons for recruitment of populations at high risk of HIV infection.

## Introduction

The World Health Organization (WHO) identifies uniformed armed personnel among some of the key populations to be focused upon in the national HIV strategic plans for several countries in sub-Saharan Africa, due to their higher risk for HIV infection compared to the general population [1]. For example, members of the uniformed armed forces in Congo were found to have higher HIV prevalence compared to the general population (3.8% versus 1.3%) [2]. Similarly, the Uganda Peoples Defence Forces (UPDF) has been listed among the most at-risk population due to high HIV incidence rates compared to the general population (3.56 per 100 person-years, 95% confidence interval [CI]: 1.49–5.52, versus 2.1 per 100 person-years, 95%CI: 1.1–3.1) [3, 4]. This is attributed to the nature of their occupation characterised by mobility and long periods of separation from their families, which predisposes them to risky sexual behaviours [5]. Members of the Uganda Police Force (UPF) are potentially likely to follow the same trends in HIV risk since they share similar operational structures as those of the army. A study conducted in Tanzania among members of the urban police force demonstrated high-risk sexual practices including low condom use, resulting in high HIV prevalence and incidence [6]. Although most of the countries in sub-Saharan Africa have implemented efforts to address HIV in the armed forces, there have been gaps noted in the amount of research in this area [7].

Evaluation of novel HIV prevention strategies (including HIV vaccine research) necessitates the recruitment of populations with presumed high exposure to HIV and high motivation to remain under study [8]. A study conducted in a population of police officers in Dar-es-Salaam, Tanzania, demonstrated they were a suitable population for HIV vaccine research due to their high HIV prevalence and high rate of willingness to participate in future vaccine trials [9]. Thus, the first HIV vaccine trial in Tanzania was conducted in a population of police officers [10]. There is surprisingly little reliable data on the prevalence and incidence of HIV among other uniformed personnel in East Africa, including the UPF, and their suitability as potential participants of HIV vaccine trials. In this paper, we describe the findings of a study to determine the acceptability and suitability of UPF personnel for future HIV vaccine trials by setting up a cohort study to estimate the recruitment and retention rates as well as HIV incidence rate and associated factors over a one-year period.

## Methods

### Study settings

The study was conducted in a population of individuals living within police barracks communities of the Uganda Police, all situated within the Kampala and surrounding Wakiso districts. These communities were chosen because they have the highest number of residents and were thought to have a good representation of the various police departments. All potential and interested volunteers from each of these communities were referred to a centrally located study clinic based at a central Kampala police barracks (one of the largest in East Africa) maternity clinic for screening, enrolment and follow-up. The clinic offers services to both police and non-police individuals living within its catchment area.

### Volunteer identification and screening

The team of study community field workers comprising of trained counsellors and mobilisers together with peer UPF community members, moved from door to door inviting volunteers from the barracks population to attend a pre-screening involving free community voluntary HIV counselling and testing regardless of whether they were police officers or not, as long as they were barracks residents. Our study population was ultimately composed of police officers from various police departments and other civilians (usually relatives of police officers) living within the different facilities. Volunteers aged 18 to 49 years who were found to be HIV-negative were given brief information about the study and asked if they were willing to enrol in the cohort requiring quarterly visits for up to one year, and given a referral note to the study clinic for enrolment. Those found to be HIV-positive on rapid testing in the community had a serum sample sent for HIV confirmatory ELISA testing at the MRC/UVRI & LSHTM laboratory in Entebbe. The confirmed HIV-positive individuals received their results within two weeks and were referred to specified HIV treatment and care centres.

At the research clinic, HIV-negative volunteers received detailed group information about the study, followed by one to one sessions where they were allowed to ask further questions about the study. The remaining volunteers provided written informed consent for the screening and enrolment procedures, which consisted of an HIV risk assessment, a baseline medical history, followed by full physical examination including a genital examination, a confirmatory repeat HIV rapid test and a serum sample collection for syphilis testing and storage, and pregnancy testing for women.

Volunteers who fulfilled the inclusion criteria: (i) aged 18–49 years, (ii) being sexually active in the past three months, (iii) willing to attend study visits and procedures, and (iv) reported to be at high-risk for HIV in the last three months (defined by: experienced a sexually transmitted infection (STI) episode/ engaged in condomless sex with new or more than one sexual partner/ reported alcohol consumption at least 3 times a week) were then invited to the study procedures. Those enrolled provided demographic information including duration of residence in the community, locator information on how they could be traced; they were treated for any medical conditions including sexually transmitted infections (STI) that had been reported or diagnosed (clinically or laboratory); they were reimbursed for transport and time spent at the clinic (approximately US$ 3); and they received an appointment card for follow-up visits. Volunteers who were found not eligible but required treatment were offered referrals to appropriate facilities.

### Follow-up procedures

A study clerk made a reminder phone call one week before the participant’s scheduled follow-up visit date. All study follow-up visit procedures were conducted at the same study clinic. At each quarterly follow-up visit, participants received HIV counselling and testing followed by a symptom-directed physical examination, pregnancy testing (females) and syndromic management of STIs if required. Participants who tested positive for HIV were referred to convenient HIV treatment and care centres. In-depth interviews on HIV risk were conducted in months 6 and 12 during study visits. Willingness to participate in future HIV vaccine trials was assessed at months 9 and 12. At month 12, participants received repeat testing for syphilis (as well as HIV). At the end of each study visit the participant’s locator/contact data were updated and participants were reimbursed for transport and time spent at the clinic. Participants who missed their appointment date were given reminder calls and informed to come within a visit window of 7 days. At the study clinic, study counsellors offered continuous free HIV counselling and testing to anyone in the community throughout the entire study period.

### Laboratory testing

Each participant provided 5ml blood serum at each visit. HIV testing was done based on the MRC/UVRI & LSHTM standardised HIV testing algorithm at both screening and follow-up visits using the Alere Determine Rapid test (Alere Medical Co Ltd. Chiba, Japan), and if positive, an aliquot form the serum sample collected the same date was taken to the central laboratory at MRC/UVRI & LSHTM in Entebbe for confirmation using two serial ELISA tests (Murex HIV-1.2.0 Ab, Diasorin UK, followed by Bioelisa Ag+Ab, Biokit, Spain). Syphilis serostatus was determined using the Treponema Pallidum Haemagglutination Assay (TPHA, Serodia-TP.PA, Fujirebio Inc, Japan) confirmed with Rapid Plasma Reagin (RPR, BD Macro-Vue, Becton Dickson & Co, USA). All TPHA positive cases confirmed with a RPR titre greater than 1:8 were considered ‘high titre active syphilis’ cases and treatment given. Urine pregnancy testing was performed using QuickVue (Quidel Corporation, USA).

### Statistical analysis

Data were captured in MS Access, 2003 (Microsoft Corporation, Redmond, WA), and analysed in Stata 15.0 (Stata Corp, College station, TX, USA). Descriptive baseline characteristics were stratified by gender. Self-reported risk behaviours and clinician-observed characteristics were summarised by months of follow-up and compared using Chi-square or Fisher’s Exact tests. The retention rate was estimated as the number of participants retained at the final visit (month 12) divided by the number of person-years of observation (PYO), expressed as a rate per 100 PYO. PYO were estimated as the difference in the dates between enrolment and last date seen at the clinic or date of HIV infection. Rate ratios and their 95% confidence intervals (CI) were used to determine factors associated with retention in univariate and multivariable analyses using Poisson regression models. Participants’ time-changing variables were treated as such in the Poisson models, allowing for intragroup correlation by use of robust standard errors since volunteers had multiple observations. In the multivariable Poisson regression models, we retained only those factors that caused a change in the rate of >20%, except for sex and age group that were included a priori.

### Ethical considerations

The study received approval from the clinic institutional review board, the Uganda Virus Research Institute Research Ethics Committee (UVRI-REC) and the Uganda National Council for Science and Technology (UNCST). Each volunteer gave written informed consent before enrolment into the study. Individuals found to be HIV-positive were referred to appropriate treatment services, including antenatal clinics providing PMTCT, and those found to have high-titre syphilis were treated according to national guidelines at the referral clinic.

## Results

### Baseline characteristics

**Fig 1:**
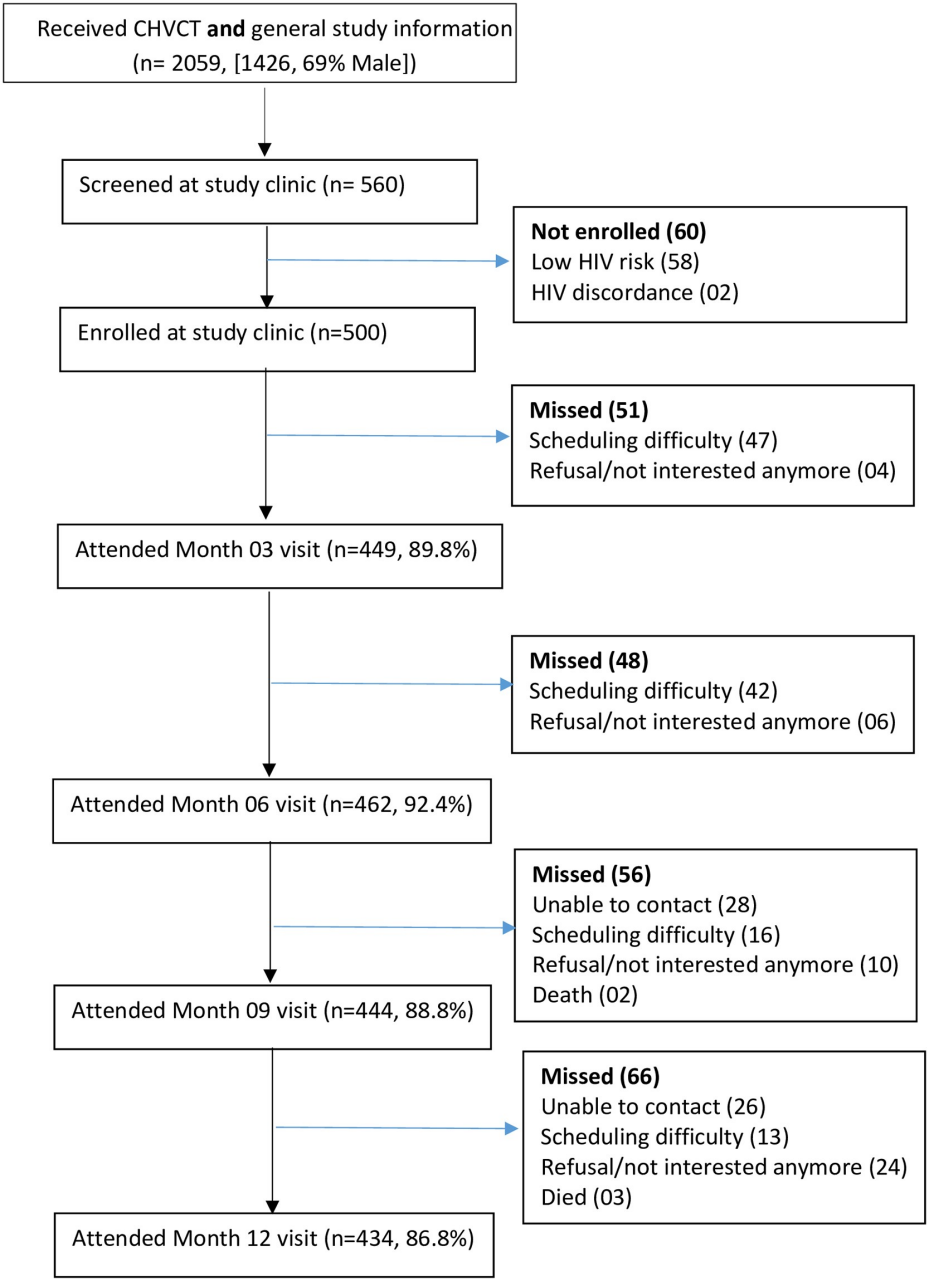
The UPF study cohort profile.

Between November 2015 and May 2016, the study team screened 560 individuals (67% males) and enrolled 500 (69% males) volunteers. Reasons for non-enrolment are presented in Fig 1, mostly because of estimated low HIV risk. Most participants were aged 25–34 years, had secondary education or more, were married, and had lived in the barracks for a period of 1 to 5 years (Table 1).

**Table 1:** Baseline characteristics of individuals recruited in the Uganda Police Force HIV vaccine preparedness study.

Characteristic	Category	Males (N = 345) n (%)	Females (N = 155) n (%)
Age group, years	18–24	81 (23.5)	53 (34.2)
	25–34	154 (44.6)	75 (48.4)
	35+	110 (31.9)	27 (17.4)
Education	Primary	19 (5.5)	39 (25.2)
	Secondary	248 (71.9)	80 (51.6)
	Tertiary	78 (22.6)	36 (23.2)
Religion	Anglican	141 (40.9)	62 (40.0)
	Catholic	149 (43.2)	51 (32.9)
	Muslim	30 (8.7)	20 (12.9)
	Pentecostal	15 (4.3)	13 (8.4)
	Other	10 (2.9)	9 (5.8)
Marital status	Single (never married)	107 (31.0)	42 (27.1)
	Married (one wife/husband)	160 (46.4)	76 (49.0)
	Married (polygamous)	42 (12.1)	7 (4.5)
	Cohabiting	32 (9.3)	27 (17.4)
	Widowed/Separated	4 (1.2)	3 (2.0)
Residence duration	< 1 year	73 (21.1)	19 (12.3)
	1–5 years	180 (52.2)	73 (47.1)
	>5 years	92 (26.7)	63 (40.6)

### HIV incidence, syphilis prevalence and incidence, and trends in reported sexual risk behavior and HIV prevention knowledge

Volunteers were followed-up for one year with the last visit occurring in May 2017. During follow-up we observed only one HIV seroconversion in 431 PYO, translating into an HIV incidence rate (IR) of 0.23/100 PYO (95%CI: 0.03–1.64). There were 4 cases of confirmed syphilis among the 500 recruited volunteers, a baseline prevalence of 0.8% (95%CI: 0.22–2.04), whilst 6 new cases of syphilis (TPHA+/RPR >1:8) were observed during follow up in 432 PYO, translating into a syphilis IR of 1.4/100 PYO (95%CI: 0.62–3.09). During follow up 31 new pregnancies were observed among the participating females over 133.7 years of follow-up resulting into a pregnancy IR of 23.19/100 PYO (95%CI: 16.31–32.97).

Over the follow-up period there was a significant reduction in the proportion of volunteers reporting high-risk behaviors, in both males and females. Amongst male volunteers there was a significant reduction in: self-reported STIs, alcohol consumption, reporting 3 or more sexual partners over the last 3 months, never using a condom with new sexual partner, travelling away from home, not practicing any HIV prevention methods. However, there were no changes in the knowledge of HIV prevention methods and male circumcision status in the cohort. For the female volunteers, there was an observed significant reduction in the reported genital ulcers and discharge, multiple partners and not practicing any HIV prevention methods. There was no observed trend in: the use of alcohol (which remained low throughout the period of follow-up compared to men), condom use with a new sexual partner, knowledge of HIV prevention and travelling away from home (see Table 2).

**Table 2:** Risk behavior at enrolment/month 0, month 6, and month 12, by sex.

Risk behavior	Males				Females			
	Month 0	Month 6	Month 12	p-value	Month 0	Month 6	Month 12	p-value
**Observed by clinician at examination**								
Genital discharge disease								
Yes	33 (9.6)	12 (3.9)	5 (2.0)	<0.01	109 (70.3)	31 (22.3)	15 (13.8)	<0.001
No	312 (90.4)	294 (96.1)	252 (98.0)		46 (29.7)	108 (77.7)	94 (86.2)	
Genital ulcers								
Yes	62 (18.0)	15 (4.9)	5 (2.0)	<0.01	92 (59.4)	18 (12.9)	7 (6.4)	<0.001
No	283 (82.0)	293 (95.1)	250 (98.0)		63 (40.6)	122 (87.1)	102 (93.6)	
Circumcision status								
Circumcised	246 (72.6)	223 (72.6)	193 (75.1)	0.746	N/A	N/A	N/A	
Not Circumcised	93 (27.4)	84 (27.4)	64 (24.9)		N/A	N/A	N/A	
**Self-report**								
Alcohol consumption								
None	134 (38.8)	148 (47.9)	141 (54.6)	<0.01	125 (80.6)	119 (85.0)	89 (82.4)	0.862*
Weekly	131 (38.0)	86 (27.8)	59 (22.9)		8 (5.2)	7 (5.0)	6 (5.6)	
1–3 times/month	57 (16.5)	62 (20.1)	48 (18.6)		20 (12.9)	14 (10.0)	13 (12.0)	
Daily	23 (6.7)	13 (4.2)	10 (3.9)		2 (1.3)	0 (0.0)	0 (0.0)	
Having sex while drunk (among those reporting drinking)								
Never	66 (31.4)	73 (46.2)	42 (36.2)	0.034*	13 (43.3)	12 (57.1)	11 (52.4)	0.811*
Sometimes	113 (53.8)	69 (43.7)	67 (57.8)		14 (46.7)	7 (33.3)	10 (47.6)	
Frequently	21 (10.0)	11 (7.0)	5 (5.3)		2 (6.7)	1 (4.8)	0 (0.0)	
Always	10 (4.8)	5 (3.1)	2 (1.7)		1 (3.3)	1 (4.8)	0 (0.0)	
Number of sexual partners								
0/1	115 (33.4)	198 (65.8)	171 (67.3)	<0.01	129 (83.2)	134 (95.7)	105 (97.2)	<0.001*
2	160 (46.5)	68 (22.6)	65 (25.6)		23 (14.8)	6 (4.3)	3 (2.8)	
3+	69 (20.1)	35 (11.6)	18 (7.1)		3 (1.9)	0 (0.0)	0 (0.0)	
Having a new sexual partner								
No	158 (46.2)	206 (76.6)	183 (82.8)	<0.01	114 (78.1)	122 (96.8)	88 (97.8)	<0.001*
Yes	184 (53.8)	63 (23.4)	38 (17.2)		32 (21.9)	4 (3.2)	2 (2.2)	
Condom use with new partner								
Never	104 (56.5)	19 (30.2)	26 (26.3)	<0.01*	15 (46.9)	2 (50.0)	1 (50.0)	0.941*
Sometimes	40 (21.7)	14 (22.2)	4 (10.5)		10 (31.3)	1 (25.0)	0 (0.0)	
Frequently	9 (4.9)	2 (3.2)	2 (5.3)		0 (0.0)	0 (0.0)	0 (0.0)	
Always	31 (16.9)	28 (44.4)	22 (57.9)		7 (21.9)	1 (25.0)	1 (50.0)	
Know any HIV prevention methods								
Yes	344 (99.7)	306 (99.0)	252 (97.7)	0.058*	153 (99.4)	140 (100)	106 (97.3)	0.097*
No	1 (0.3)	3 (1.0)	6 (2.3)		1 (0.6)	0 (0.0)	3 (2.7)	
Practice any HIV prevention method								
Yes	295 (85.8)	295 (96.4)	231 (91.7)	<0.01	142 (92.8)	140 (100)	104 (98.1)	0.001*
No	49 (14.2)	11 (3.6)	21 (8.3)		11 (7.2)	0 (0.0)	2 (1.9)	
HIV prevention methods practiced								
Condom	187 (63.4)	111 (37.4)	73 (30.8)	<0.01*	20 (14.1)	13 (9.3)	7 (6.5)	0.003*
Being Faithful	82 (27.8)	157 (52.8)	146 (61.6)		111 (78.2)	121 (86.4)	90 (84.1)	
Abstinence	16 (5.4)	19 (6.4)	18 (7.6)		4 (2.8)	6 (4.3)	10 (9.4)	
Other	10 (3.4)	10 (3.4)	0 (0.0)		7 (4.9)	0 (0.0)	0 (0.0)	
Travelling								
Yes	259 (76.2)	199 (64.6)	146 (56.8)	<0.01	42 (29.0)	32 (22.9)	24 (22.0)	0.354
No	81 (23.8)	109 (35.4)	111 (43.2)		103 (71.0)	108 (77.1)	85 (78.0)	

*Fisher’s Exact p-value

### Retention and associated factors

Over the period of follow up, 376 volunteers completed their final follow-up, occurring over a period of 432 PYO, resulting in an overall retention rate of 87% (Table 3). In the adjusted model, retention at one year was significantly associated with the following baseline characteristics: residence duration (higher among those who had resided for 1 to 5 years (adjusted rate ratio [aRR] = 1.19, 95%CI: 1.00–1.44) or had spent more than 5 years (aRR = 1.34, 95%CI: 0.95–1.37) compared to those who had resided for less than a year); absence of genital discharge in the last 3 months (higher among those who reported no genital discharge (aRR = 1.97, 95%CI: 1.38–2.83) compared to those reporting genital discharge); absence of genital ulcers in the last 3 months (higher among those reporting no ulcers (aRR = 1.90, 95%CI: 1.26–2.87) compared to those reporting ulcers); alcohol consumption in the last month (lower retention among those reporting daily consumption (aRR = 0.68, 95% CI: 0.41–1.11) compared to those who had not drank alcohol); report of a new sexual partner in the last month (lower in those reporting new partner (aRR = 0.57, 95%CI: 0.45–0.71) compared to those reporting no new partner), time away from home, higher among those away for more than two nights (aRR = 1.27, 95%CI: 1.04–1.56) compared to those who had no overnight trips in the preceding period) and absence of knowledge on HIV prevention (aRR = 2.67, 95%CI: 1.62–4.39) compared to those who reported knowledge(Table 3).

**Table 3:** Univariate and multivariable analyses of factors associated with one-year retention in a cohort of Uganda Police Force community in Kampala and Wakiso Districts, Uganda.

Variable	Attended	PYO	Rate	RR (95%CI)	LRT P-value	aRR (95%CI)
**Overall 1 year retention**	376	432.1	87.0			
**Gender**						
Male	261	296.9	87.9	1	0.506	1
Female	115	135.2	85.1	0.97 (0.88–1.07)		0.87 (0.75–1.01)
**Age, years**						
18–24	96	114.3	84.0	1	0.472	1
25–34	173	199.2	86.8	1.03 (0.92–1.16)		1.06 (0.92–1.23)
35+	107	118.2	90.3	1.08 (0.95–1.21)		1.14 (0.98–1.33)
**Religion**						
Muslim	29	38.8	74.8	1	<0.001	
Catholic	154	175.6	87.7	1.17 (0.95–1.45)		
Anglican	155	175.7	88.2	1.18 (0.96–1.45)		
Pentecostal	20	25.5	78.5	1.05 (0.77–1.43)		
Other (Seventh day Adventist)	18	16.5	100	1.46 (1.20–1.78)		
**Education level**						
Primary	43	52.0	82.7	1	0.768	
Secondary	244	161.6	87.6	1.06 (0.91–1.24)		
Tertiary	89	101.6	87.6	1.06 (0.89–1.26)		
**Marital status**						
Single	214	244.8	87.4	1	0.963	
Married	51	58.5	87.1	0.99 (0.87–1.14)		
Widowed/Separated	111	128.7	86.2	0.99 (0.89–1.09)		
**Duration of residence**						
Under 1 year	59	79.1	74.6	1	0.050	1
1 to 5 years	195	216.3	90.2	1.21 (1.04–1.41)		**1.19 (1.00–1.44)**
> 5 years	122	136.8	89.2	1.20 (1.02–1.40)		1.34 (0.95–1.37)
**Genital discharge, last three months**						
Yes	25	59.3	42.2	1	<0.001	1
No	351	372.8	94.1	2.23 (1.60–3.11)		**1.97 (1.38–2.83)**
**Genital ulcers, last three months**						
Yes	21	55.8	37.6	1	<0.001	1
No	355	376.3	94.4	2.51 (1.72–3.66)		**1.90 (1.26–2.87)**
**Circumcision (males only)**						
Yes	199	216.5	91.9	1	0.091	
No	62	78.8	78.6	0.86 (0.71–1.03)		
**If not, do you intend to get circumcised (males only)**						
Yes	75	65.2	100	1	0.001	
No	8	15.6	51.5	0.45 (0.25–0.81)		
Not sure	6	12.8	46.8	0.41 (0.20–0.81)		
**Drank alcohol, last month**						
None	236	249.0	94.8	1	0.007	1
1–3 times a month	60	72.0	83.3	0.88 (0.71–1.09)		0.92 (0.73–1.15)
Weekly	70	97.1	72.1	0.76 (0.64–0.91)		**0.74 (0.61–0.91)**
Daily	10	14.0	71.5	0.75 (0.471.20)		0.68 (0.41–1.11)
**Alcohol consumption before sex, last month**						
Never	144	156.2	92.2	1	0.280	
Sometimes < than half the time	193	224.0	86.2	0.93 (0.81–1.07)		
Frequently > than half the time	28	35.3	79.3	0.86 (0.67–1.11)		
Always	11	16.7	66.1	0.72 (0.47–1.10)		
**Number of sexual partners, last month**						
0–1	284	295.9	95.9	1	0.001	1
2	73	96.2	75.8	0.79 (0.65–0.97)		0.90 (0.73–1.12)
3	19	39.8	47.7	0.50 (0.34–0.73)		0.74 (0.48–1.12)
**New sexual partner, last 3 months**						
No	317	318.8	99.4	1	<0.001	1
Yes	59	113.2	52.1	0.52 (0.42–0.65)		**0.57 (0.45–0.71)**
**Condom with new partner, last 3 months**						
Never	10	7.5	100	1	0.296	
Sometimes < than half the time	3	5.4	56.0	0.42 (0.13–1.35)		
Frequently > than half the time	2	0.9	100	1.67 (0.59–4.68)		
Always	20	13.2	100	1.14 (0.66–1.95)		
**Away from home ≥ 2 nights, last 3 months**						
Yes	181	235.1	77.0	1	0.003	1
No	195	197.0	99.0	1.29 (1.09–1.52)		**1.27 (1.04–1.56)**
**Do you know any HIV prevention method**						
Yes	367	427.4	85.9	1	0.017	1
No	9	4.7	100	2.45 (1.16–4.35)		**2.67 (1.62–4.39)**
**Do you practice any HIV prevention method**						
Yes	355	404.7	87.0	1	0.499	
No	21	27.3	76.9	0.88 (0.60–1.28)		
**If yes, current prevention practices**						
Abstinence	24	24.7	97.3	1	<0.001	
Being faithful to my partner	257	243.2	100	1.09 (0.77–1.53)		
Condom	86	136.3	63.1	0.65 (0.44–0.95)		
Other	7	10.1	69.3	0.71 (0.40–1.27)		

## Discussion

Our study shows that it is feasible to recruit and adequately follow up volunteers from a population composed of police force and their relatives for research. We established incidence rates of HIV and syphilis in this population. However, the data show an unexpectedly low HIV incidence and low syphilis prevalence which might be as a result of our recruitment methods. During recruitment some individuals at high risk of infection were selected out at screening and lost to follow up. The heterogeneous composition of our cohort, including non-police officers could have caused some form of risk dilution by including low risk individuals explaining the low incidence if HIV despite high rates of condomless sex as evidenced, for women, by the high pregnancy rates. Of the 2059 individuals who attended the community voluntary HIV testing, only 560 (27.2%) made it to the clinic for further study eligibility assessment. This was because majority of those approached only required HIV testing services and although the study sample size of 500 was attained, the testing service continued.

The observed low incidence of HIV and syphilis is encouraging and may be partly due to the education level and prior HIV/STI -prevention knowledge of those recruited into the cohort. In addition, HIV testing is mandatory for recruitment into the police force in Uganda, and only HIV negative individuals are eligible to stay, this is likely to have contributed to the low prevalence and incidence because we had a number of newly recruited members of the force in the cohort. Of note, the low HIV incidence would make this population suitable for phase I and II trials which require low risk individuals for safety and immunogenicity studies [11].

The observed decline in the reported risk behaviours over one year could be attributed to the risk reduction counselling that was offered at each quarterly visit. A similar finding was observed in a female sex worker cohort study, by Traore et al. [12] in Burkina Faso, where zero HIV infections were observed following a combination intervention over a two year period. In another study conducted by Kaul et al. [13] among female sex workers in Nairobi, a reduction in risk taking was observed after an intensive period of risk reduction counselling and regular STI treatment. In another study, Ghys et al. [14] found that HIV prevention intervention contributed to significant lowering of the HIV-1 seroincidence rate during the intervention study than before the study (6.5 versus 16.3 per 100 person-years; P = 0.02). However, such a reduction as observed in our study should be interpreted with caution, since it could also be a result of social desirability bias [15, 16].

Our study demonstrated a good overall retention rate in this population. In common with similar studies [17], we observed that retention was better among volunteers who had lived in the facility longer, a possible reflection of their relative stability, which may also have affected their HIV-infection risk. Volunteers who reported no knowledge of HIV risk were more likely to be retained compared to those reporting to be knowledgeable, possibly because the latter sought to attend in order to acquire more knowledge. We observed that loss to follow up was associated with volunteers who reported the most high-risk behaviours as well as those who reported having travelled away from home in the last month. This association may also explain the low incidence observed in the study.

Our study had limitations. Firstly, during screening and recruitment of our study population, we did not systematically recruit to ensure a weighted representation from the different police departments and ranks, giving rise to possible selection bias. From our anecdotal observations, we noted that the majority of enrolled police officers were from lower ranks, and we had no representation from some of the departments such as the traffic and mobile patrol units, who might differ in terms of the variables and outcomes we were investigating. Secondly, our study was not designed to collect specific reasons why volunteers were lost to follow up, which would be useful in explaining the reasons and so inform possible interventions. Thirdly, the study findings are based mostly on self-report which might potentially introduce social desirability bias if participants choose to modify their responses to mask risk behaviour. However, inclusion of biological information such as HIV and syphilis incidence add credence to our findings.

## Conclusions

The study showed it is possible to recruit and adequately follow up volunteers from the community of the Uganda Police Force for participation in future HIV vaccine trials. The low HIV incidence and decline in HIV risk behaviour during follow-up, combined with the favourable retention rate could make this population potentially suitable for Phase I & II HIV vaccine trials, where low risk individuals are required. However, the surprisingly low HIV incidence in our cohort suggests that those at higher risk of infection (i.e., those in mobile divisions in the force) may have been omitted from our cohort, and such population would not be adequate for Phase III HIV vaccine trials. We recommend more stringent sampling to ensure greater representation of different ranks and divisions (i.e. traffic police) in future epidemiological studies in such similar populations.
